# Simple dietary advice targeting five urinary parameters reduces urinary supersaturation in idiopathic calcium oxalate stone formers

**DOI:** 10.1007/s00240-020-01194-7

**Published:** 2020-06-10

**Authors:** Juri Sromicki, Bernhard Hess

**Affiliations:** 1 Internal Medicine and Nephrology, Kidney Stone Center Zurich, Klinik Im Park and University of Zurich, Bellariastrasse 38, CH-8038 Zurich, Switzerland; 2 Department of Cardiac, Vascular and Thoracic Surgery, University Hospital, CH-8091 Zurich, Switzerland

**Keywords:** Idiopathic calcium oxalate stone formers, Dietary advice, Urinary supersaturation

## Abstract

Among 208 kidney stone patients referred within 2 years, 75 patients (66 men, nine women) with truly idiopathic calcium oxalate stones (ICSF) were recruited. Dietary advice (DA) aimed at (1) urine dilution, (2) reduced crystallization promotion (lowering oxalate), and (3) increased crystallization inhibition (increasing citrate). We recommended higher intakes of fluid and calcium with meals/snacks (reducing intestinal oxalate absorption) as well as increased alkali and reduced meat protein (acid) for increasing urinary citrate. The intended effects of DA were elevations in urine volume, calcium (U-Ca) and citrate (U-Cit) as well as reductions in oxalate (U-Ox) and uric acid (U-UA). We retrospectively calculated an adherence score (AS), awarding + 1 point for parameters altered in the intended direction and − 1 point for opposite changes. Calcium oxalate supersaturation (CaOx-SS) was calculated using Tiselius’ AP(CaOx) index EQ. DA induced changes (all *p* < 0.0001) in urine volume (2057 ± 79 vs. 2573 ± 71 ml/day) and U-Ca (5.49 ± 0.24 vs. 7.98 ± 0.38 mmol/day) as well as in U-Ox (0.34 ± 0.01 vs. 0.26 ± 0.01 mmol/day) and U-UA (3.48 ± 0.12 vs. 3.13 ± 0.10 mmol/day). U-Cit only tendentially increased (3.07 ± 0.17 vs. 3.36 ± 0.23 mmol/day, *p* = 0.06). DA induced a 21.5% drop in AP(CaOx) index, from 0.93 ± 0.05 to 0.73 ± 0.05 (*p* = 0.0005). Decreases in CaOx-SS correlated with AS (*R* = 0.448, *p* < 0.0005), and highest AS (+ 5) always indicated lowering of CaOx-SS. Thus, simple DA can reduce CaOx-SS which may be monitored by AS.

## Introduction

Because recurrence rates in calcium oxalate stone formers may reach 50% within 10 years [1], prophylactic measures are indicated. They include either clinical observation and immediate urological treatment of recurrences, or comprehensive metabolic evaluation and preventive treatment [2]. The latter consists of dietary advice and/or medication. For many years, our daily clinical approach in idiopathic calcium oxalate stone formers has been to reduce urinary supersaturation primarily by dietary advice (DA) alone, based on explanations of stone forming mechanisms and individual dietary preferences of patients [3].

The ultimate goal of any DA should be to reduce urinary supersaturation, the driving force for stone formation [4]. Indeed, Prochaska et al. [5] have recently demonstrated in large cohorts that the likelihood of becoming a stone former increases almost linearly with increasing calcium oxalate supersaturations (CaOx-SS), both in women and in men. Although dietary effects on overall stone risk are mentioned in many guidelines and review articles, only few prospectively controlled investigations have addressed the direct effect of dietary modifications alone (without medication) on urine supersaturations [6–9]. Only one short 7-day study [6], taking into account multiple dietary factors according to the Guidelines of the European Association of Urology [10], achieved significant reductions in urinary supersaturations of calcium oxalate as well as brushite and uric acid, measured by EQUIL 2 [11]. Two other, more extended studies applied either a DASH-style diet (high in fruit, vegetables, whole grains and low-fat dairy products, low in saturated fat, total fat, cholesterol, refined grains, sweets and meat) [7] or diets low in animal protein vs. high in fiber [8]. Overall, all these protocols were not able to significantly lower urinary CaOx-SS [7, 8].

Our own study in idiopathic calcium stone formers [9], using EQUIL 2 for supersaturation calculations [11], had a mean follow-up of 5 months and compared CaOx-SS before and after advice for a “common sense diet”. The advice aimed at high urine volume (> 2000 ml/day), lower daily meat protein (about 1 g/kg body weight) and lower sodium intake (24-h urine sodium < 200 mmol/day) as well as daily consumption of 800 mg calcium from dairy products. Overall, CaOx-SS remained unchanged on the recommended diet. Interestingly, individual diet-induced increases in CaOx-SS were strongly positively related to increases in urinary oxalate, but not to rises in urinary calcium [9]. On the other hand, CaOx-SS significantly decreased with increases in urine volume, urine pH, and gastrointestinal alkali intake [9]. This is in accordance with Robertson [4] who described low urine volumes, high urinary oxalate and lack of urinary citrate (alkali) as major drivers of CaOx-SS and crystallization.

Whereas increasing urine volume has emerged as a logic prophylactic measure for lowering urine supersaturation, it is still not entirely clear how the important reduction in urinary oxalate is best achieved. Many physicians still recommend a diet low in oxalate. However, as summarized by Robertson [4], there is to date no evidence from controlled trials that decreasing oxalate intake has a beneficial effect on stone recurrences. Moreover, physical chemistry predicts that even small increases in urinary oxalate are much more dangerous for CaOx-SS than rises in urinary calcium [12], because there is always a considerable molar excess of calcium present in urine (for instance, from nutrition and bone metabolism), whereas calcium oxalate crystallization occurs in a 1:1 molar ratio. Thus, small increases in urinary oxalate, for instance, after oxalate-containing snacks, will immediately promote calcium oxalate crystallization, because of the excess calcium ions available for crystallizing with oxalate in urine. On this background, we have demonstrated in humans that the severe hyperoxaluria occurring after ingestion of a 20-fold amount of oxalate can be abolished by simultaneous ingestion of large amounts of calcium from natural sources [13]. This allows calcium oxalate precipitation already in the intestinal tract, whereby enteric absorption and urinary excretion of oxalate are reduced [13]. Finally, reducing animal protein consumption (meat, fish, poultry) and increasing intake of alkali (vegetables, salad, fruit) reduce hyperacidity and raise urinary citrate, a chelator of calcium ions [14]. These changes reduce urinary CaOx-SS as driving force for stone formation [4, 14, 15].

In the present study, we tested the effects of a simple DA on 24-urinary chemistries and CaOx-SS in truly idiopathic calcium oxalate stone formers (ICSF). Our DA primarily addresses five parameters which appear pathophysiologically most relevant to CaOx supersaturation and stone formation, namely urine volume, calcium (U-Ca) and oxalate (U-Ox) as well as uric acid (U-UA, marker of “acid”, i.e., animal protein) and citrate (U-Cit, marker of alkali). Changes in urine chemistries were expressed by a simple, retrospectively assigned adherence score in every single ICSF, and alterations in CaOx-SS were correlated with changes in urine chemistries as well as with the adherence scores.

## Subjects and methods

### Stone clinic setup and study subjects

This study was performed in a small private nephrology/internal medicine practice setup (one nephrologist/internist, two nurses/secretaries) which receives regular referrals of kidney stone patients from practicing urologists, mainly from the greater Zurich area. Among 208 kidney stone patients referred for metabolic evaluation and stone recurrence prophylaxis to our stone clinic in 2017 and 2018, we selected those with truly idiopathic calcium oxalate stones, based on patients’ history and results of stone analyses (X-ray diffraction or infrared spectroscopy) provided by referring urologists or obtained from stone specimens delivered by the patients. Thus, we excluded all stone formers with pure or mixed stones containing apatite, brushite, uric acid, cystine and struvite as well as those with short bowel syndrome (bariatric surgery, inflammatory bowel disease), patients with distal renal tubular acidosis (incomplete or complete), patients with hyperparathyroidism or sarcoidosis, and all stone formers in need of medication (primarily alkali citrate, thiazides and allopurinol). We were finally able to study 75 truly idiopathic stone formers (ICSF) with pure calcium oxalate stones and at least one follow-up urine collection after basal evaluation.

## Methods

### Laboratory investigations

In accordance with our standard protocol published previously [3, 16], fasting venous blood was drawn and a fasting urine sample (second morning urine after 12 h of fasting) as well as two 24-h urines were collected under free-choice diet. Twenty-four hour urines were collected in 3-L plastic containers containing 10 g of boric acid which does not affect the concentration measurements of urinary constituents and pH [16]. These urines were analyzed for volume, pH, creatinine, protein, sodium, potassium, calcium, uric acid, phosphate, urea, and magnesium by autoanalyzer techniques [3, 16]. Oxalate and citrate were measured by ion chromatography (UNILABS, Dübendorf, Switzerland). The current study exclusively focused on results from 24-h urine collections delivered during basal metabolic evaluation (values are means of two 24-h urine collections) and in one follow-up 24-h urine collected at least 3 months after dietary advice.

Urinary supersaturation with respect to calcium oxalate (CaOx-SS) was calculated using the AP(CaOx) index EQ, developed by Tiselius [17] as follows:

$$\text{AP} \, \left( {\text{CaOx}} \right) \, \text{index} \, \text{EQ} = 1.9 \times \text{Ca}^{0.84} \times \text{Ox}{\text{ x }}\text{Mg}^{{ - }^{0.12}} \times \text{Cit}^{{ - }^{0.22}} \times \text{V}^{{ - }^{1.03}}.$$

These calculations are easier to perform than the more elaborate EQUIL program, and the index shows an excellent linear correlation with the “gold-standard” EQUIL 2 (*R* value 0.98) [17].

### Dietary history and dietary advice (DA)

All patients were asked to write down as precisely as possible the dishes and beverages that they were consuming during the 24-h urine collection periods. Individual dietary protocols were screened for fluid intake, types of beverages, whether oxalate-rich products were consumed separately or simultaneously with calcium (mainly mineral waters), number of meat protein servings per day, number of servings of alkali-rich products (vegetables, salad, fruit) and calcium-rich products (mineral waters, dairy products). In addition, a detailed dietary history was taken (always obtained by BH), whereby patients were systematically asked to estimate their daily intakes of the following items: total fluid intake (all beverages, soup), any intake of oxalate-rich products during main meals and snacks (i.e., black and green tea, cereals, oatmeal, bran products, wheat-chocolate energy bars, chocolate, chocolate biscuits, nuts/peanuts, almonds, pistachios, olives, chard, spinach, rhubarb, beetroot, beans, whole grain bread, tomatoes, blueberries and cranberries), meat protein (all meat/sausage, fish, poultry, deer), alkali (vegetables, salad, fruit), and calcium (natural sources and supplements). Furthermore, intakes of calcium, salt, and protein were always assessed as follows:

- Calcium intake from mineral waters and dairy products, the main sources of calcium, was semiquantitatively assessed by using publicly available tabulated data of the calcium content of mineral waters and various dairy products (milk, yogurt, curd cheese, various sorts of cheeses) regularly consumed in Switzerland. The total amount of calcium per week, as derived from individual patient’s information, was divided by seven to obtain a mean daily calcium intake.
- Salt consumption was calculated based on the 24-h urine excretion of sodium and the molecular weight of NaCl (58.4 g/mol), using the formula
  - Urine −  Na (mmol/day) × 0.0584 = g salt/day.
- Total protein consumption was calculated based on 24 h urine excretion of urea by the formula
  - [Urine −  Urea × 0.18] + 13 = g protein/day [18]
  - normalized to kilograms normal body weight (Height − 100).

On the basis of this information as well as on stone analysis and urine chemistries, DA for prophylaxis of stone recurrences was provided (always by BH). The general recommendations were always as follows:

- Dilution: greatly increase fluid intake, i.e., urine should permanently be light yellow to reach a urine volume of at least 2 L/day [19]. Especially, drinking under stress (for instance, at work, hot environments) and during the night was emphasized.
- Reduce crystallization promotion: reduce U-Ox by increasing calcium intake. Previously, we had been able to normalize hyperoxaluria on a 20-fold oxalate intake (2220 mg = 24.7 mmol/day) in humans, when calcium intake was increased from 1200 to 3859 mg/day (96.5 mmol/day), i.e., with a calcium/oxalate molar ratio in the diet of 3.9/1 [13]. For the sake of simplicity under everyday practice conditions in this study, we advised stone formers to ingest equal amounts of calcium and oxalate during all meals and snacks. Patients received lists of the contents of calcium (calcium-rich mineral waters and dairy products) as well as oxalate (most important oxalate-containing products), both in beverages and nutrients. Assuming that all calcium and oxalate was soluble in the intestinal tract and patients ingested calcium and oxalate as recommended in a 1:1 ratio, the intestinal molar ratio of calcium (molecular weight 40) to oxalate (molecular weight 90) would be 2.25/1. This would mean that enough calcium was available for intestinal oxalate precipitation as well as for the overall calcium supply of the body. Since oxalate is present in all plant-derived products and thus is basically unavoidable on free-choice diet, patients were simply instructed to always ingest calcium simultaneously with any snack or meal. This is mainly achieved using calcium-rich Swiss mineral waters (calcium content > 400 mg/L) or milk as beverages with meals and snacks. Furthermore, a total calcium intake of 1200 mg/day was prescribed, because this had been shown to significantly reduce U-Ox and stone recurrence rates over 5 years in patients with idiopathic calcium oxalate nephrolithiasis in comparison with a low-calcium diet [20]. In the few patients who were not able/willing to ingest 1200 mg of calcium/day from natural sources alone, supplements (500 mg of calcium per dose) were additionally prescribed.
- Improve crystallization inhibition: increase U-Cit by reducing intake of “acid” and increasing consumption of “alkali”, as mentioned above [4, 14, 15]. “Acid”, i.e., animal protein (defined above), was instructed to be limited to one serving per day. Specifically, U-UA was taken as a marker of animal (non-dairy) protein, because in comparison with other urinary protein markers such as phosphate and sulfate, only uric acid increases significantly on certain acid-rich diets in humans [21]. “Alkali”: patients were taught to permanently eat a minimum of three portions of vegetables, salad, and fruit per day to increase U-Cit.

### 24-h urine parameters and adherence scores

For several years, all stone formers going through metabolic evaluation in our stone clinic [3] have to collect another 24-h urine 3 months after basal evaluation and DA. According to the main dietary changes recommended, as mentioned above, the evaluation of follow-up 24-h urines in comparison with basal 24--h urine chemistries in daily clinical practice focused primarily on the following five urine parameters:

- 1. Dilution: did urine volume rise?
- 2. and 3. Reduce promotion: did U-Ca rise (as a consequence of increased calcium intake) and, therefore, U-Ox drop (reduced gastrointestinal absorption due to intestinal CaOx precipitation)?
- 4. and 5. Improve inhibition: did U-UA (marker of animal protein) drop and U-Cit (marker of alkali) rise?

It is well known that normal values of urinary parameters relevant to stone disease are not only somewhat arbitrary, but strongly depend on specific populations and their local nutritional habits. For this reason, we had originally obtained normal 24-h urine values among 103 male and 73 female healthy Swiss volunteers consuming a habitual Swiss diet [16]. According to these normal values, thresholds of the five relevant parameters in the present study are as follows: U-Ca ≤ 9.00 mmol/day and ≤ 8.00 mmol/day in men and women, respectively, U-Ox ≤ 0.440 mmol/day (both genders), U-UA ≤ 5.00 mmol/day and < 4.00 mmol/day in men and women, respectively, and U-Cit ≥ 1.70 and ≥ 1.90 mmol/day, in men and women, respectively [16]. Because stone risk increases exponentially with urine volumes below 2 L/day [4], we consider a normal urine volume in stone formers to be at least 2000 ml/day.

In our stone center, where calculations of urinary supersaturations are not routinely performed, we clinically always focus on changes of these five urinary parameters in our discussions with patients. Thus, we retrospectively calculated an adherence score as a sort of internal quality control for quantifying the extent of adherence to our DA which can help to identify targets of further treatment for every single ICSF. For all five urinary parameters mentioned above, we calculated differences between values of the follow-up 24-h urine minus respective values during basal evaluation (mean of two urine collections). If a difference, regardless of its extent, indicated that a parameter had been altered in the intended direction (for instance an increase in urine volume after DA), we awarded + 1 point. On the other hand, we deducted one point if a parameter had changed towards a higher stone risk (for instance if urine oxalate increased in a follow-up urine). Thus, a maximum of five points (all five parameters improved) and a minimum of − 5 points (all parameters worsened) could be obtained. If diet history and follow-up urine parameters indicated that a patient had difficulties in following consequently the provided recommendations (for, instance problems with constantly drinking calcium-rich beverages simultaneously with meals), extra 24-h urine collections were performed another 3 months after reinforcing DA, until the patient had reached his best possible adherence score or had decided not to come back for further 24-h urine controls. For statistics, the patients’ best follow-up 24-h urine collections (i.e., highest adherence scores) were always used.

### Statistics

All values are means ± SEM. Paired *t *test was used to compare urine parameters before and after DA. Linear regression analysis was performed for correlations of changes in urinary CaOx-SS with single urinary parameters as well as with adherence scores, using the IBM SPSS statistics for Windows, Version 25 (IBM Corp., Armonk NY, USA).

## Results

### Stone formers’ characteristics, basal 24-h urine abnormalities and stone types

Table 1 summarizes the basal characteristics of the 75 truly idiopathic calcium oxalate stone formers: they were mainly males, and the mean age was 51.5 ± 1.5 years (range 18–73 years). The mean number of stone episodes was 6.5 ± 0.8, with 67 patients being recurrent stone formers (between 2 and 40 stone episodes). Arterial hypertension (25% of all stone formers), dyslipidemia (16%), Vitamin D deficiency (15%) and hyperuricemia (12%) were the four most frequent main comorbidities. Further main comorbidities occurring in more than one patient were osteoporosis/osteopenia (7%), metabolic syndrome (5%) and diabetes mellitus type 2 (3%). As depicted in Table 1, the majority of patients (61%) had previously been advised to or self-selectedly followed a low-calcium diet. This was reflected in the semiquantitatively obtained calcium intake (see “Methods”) from mineral waters and dairy products which only amounted to 371 ± 29 mg/day on low-calcium diet vs. 994 ± 104 mg/day on unrestricted calcium intake, *p* < 0.0001. Average salt intake was 10 g/day, and total protein intake (based on urinary urea excretion) was 83 g/day.

**Table 1:** Basal characteristics of 75 truly idiopathic calcium oxalate stone formers

Parameter	
Gender	66 men/9 women
Age (years)	51.5 ± 1.5
Weight (kg)	83.6 ± 1.8
BMI (kg/m^2^)	27.2 ± 0.5
Number of stone episodes	6.5 ± 0.8
Main comorbidities	
Arterial hypertension	19/75 (25.3%)
Dyslipidemia	12/75 (16.0%)
Vitamin D deficiency	11/75 (14.7%)
Hyperuricemia	9/75 (12.0%)
Low-calcium diet (self-selected or recommended)	46/75 (61.3%)
Daily calcium intake (mg) on low-calcium diet (*n* = 46 )	371 ± 29
Daily calcium intake (mg) without restriction (*n* = 29)	994 ± 104*
Salt intake (g/day)	10.1 ± 0.4
Total protein intake (g/day)	83 ± 2

Calcium intake reflects calcium consumption from dairy products and mineral waters, as indicated by the patients and calculated from publicly available data in Switzerland. Salt and total protein intake was derived from 24-h urine excretions of sodium and urea, respectively. For details, see text

*p < 0.001 vs. habitual low calcium intake

**Fig. 1:**
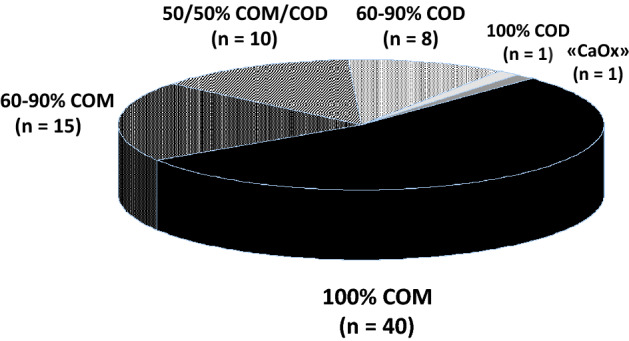
Stone analyses in 75 truly idiopathic calcium oxalate stone formers. *COM* calcium oxalate monohydrate, *COD* calcium oxalate dihydrate. Proportions of COM and COD in stones are depicted in percent of crystalline stone material, as provided from the various analytical laboratories

Based on basal 24-h urine analyses (mean of two collections), 49.3% (37/75) exhibited low urine volume, 13.3% (10/75) hyperoxaluria, 12.0% (9/75) hypocitraturia, 8.0% (6/75) hyperuricosuria, and 4.0% (3/75) hypercalciuria. The results of stone analyses are depicted in Fig. 1. Pure (100%) calcium oxalate monohydrate (COM) was the most frequently observed stone type, whereas pure calcium oxalate dihydrate (COD) was only present in one patient. In a single patient, stone analysis just reported “calcium oxalate” without differentiation between mono- and dihydrate.

### 24-h urine chemistries before and after dietary advice

Table 2 shows the changes in urinary parameters following DA. All five parameters that were considered important changed in the intended direction: urine volume increased by 25%, from 2057 ± 79 to 2573 ± 71 ml/day, and urinary calcium by 47%, and from 5.49 ± 0.24 to 7.98 ± 0.38 mmol/day. On the opposite, urinary oxalate dropped by 21%, from 0.34 ± 0.01 to 0.23 ± 0.01 mmol/day. This was also the case for urinary uric acid which fell by 10%, from 3.48 ± 0.12 to 3.13 ± 0.10 mmol/day. These changes were all statistically significant (*p* < 0.0001).

**Table 2:** Urinary parameters before and after dietary advice (DA)

Urine parameter	Before DA	After DA	*p* value
Volume (ml/day)	2057 ± 79	2573 ± 71	< 0.0001
pH	5.77 ± 0.05	5.78 ± 0.05	0.87
Creatinine (mmol/day)	13.7 ± 0.4	13.3 ± 0.4	0.15
Sodium (mmol/day)	174 ± 7	184 ± 8	0.19
Potassium (mmol/day)	65 ± 2	70 ± 3	0.1
Calcium (mmol/day)	5.49 ± 0.24	7.98 ± 0.38	< 0.0001
Oxalate (mmol/day)	0.34 ± 0.01	0.26 ± 0.01	< 0.0001
Urea (mmol/day)	389 ± 11	383 ± 14	0.91
Phosphate (mmol/day)	29.1 ± 0.9	27.6 ± 1.2	0.25
Uric acid (mmol/day)	3.48 ± 0.12	3.13 ± 0.10	< 0.0001
Citrate (mmol/day)	3.07 ± 0.17	3.36 ± 0.23	0.06
Magnesium (mmol/day)	4.38 ± 0.14	5.41 ± 0.23	< 0.0001
AP (CaOx) index EQ	0.93 ± 0.05	0.73 ± 0.05	0.0005

Data before DA are means of two 24-h urine collections. For details, see text

Urinary citrate, which positively related to U–K as a marker of alkali (*R* = 0.375, *p* = 0.001), only tended to increase, from 3.07 ± 0.17 to 3.36 ± 0.23 mmol/day (*p* = 0.06). This could be explained by the fact that patients were advised to increase their calcium intake from mineral waters as well as from dairy products. The latter is another source of protein and, therefore, conveys an additional acid load [22]. Thus, when we separately compared urinary urea as a combined marker of meat as well as dairy protein before and after DA, the excretions were virtually unchanged (389 ± 11 before vs. 383 ± 14 mmol/day after DA), as were values of protein intakes per kg normal body weight (1.11 ± 0.02 g/kg before and 1.11 ± 0.03 g/kg after DA, *p* = 0.87). This appears to indicate that patients had moved away from animal non-dairy protein (reduction in uric acid excretion) to a somewhat higher intake of dairy protein as a source of calcium. This most likely resulted in a less than expected reduction in overall acid load which explains the marginally significant increase in U-Cit. Neither U-Na nor U-K changed after DA, and U-Cit remained significantly correlated with U-K also after DA (*R* = 0.394, *p* < 0.0005).

### Urinary supersaturation with respect to calcium oxalate

As calculated by the AP(CaOx) index EQ from Tiselius [17], urinary CaOx-SS dropped by 21.5%, from 0.93 ± 0.05 to 0.73 ± 0.05, *p* = 0.0005. Numerically, AP(CaOx) index EQ fell in 52 out of 75 stone formers (69%) after DA. When we correlated the decrease in AP(CaOx) index EQ to changes in all five urine parameters (always value before minus value after dietary advice), it was inversely related to the change in urine volume (*R* = − 0.496, *p* < 0.0005), i.e., the more urine volume increased, the more AP(CaOx) index EQ decreased. On the other hand, the decrease in AP(CaOx) index EQ after DA was positively correlated to decreases in U-Ox (*R* = 0.562, *p* < 0.0005) and weakly to U-UA (*R* = 0.237, *p* = 0.041), i.e., decreases in supersaturation most strongly depended on decreases in U-Ox and increases in urine volume. However, no significant correlation was found between the decrease in AP(CaOx) index EQ and changes in U-Ca and U-Cit.

### Adherence scores

**Fig. 2:**
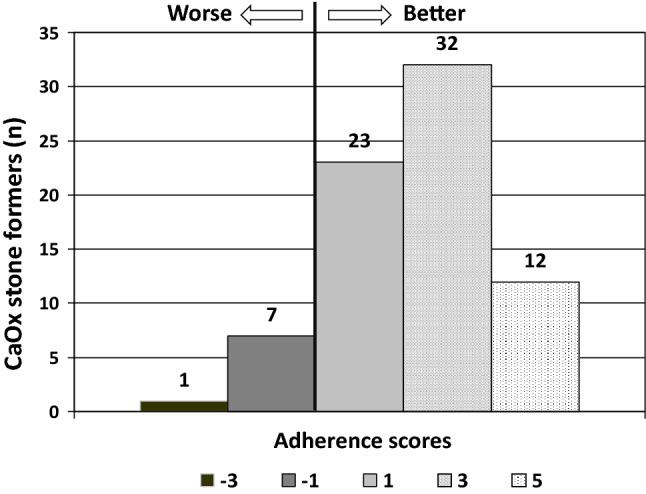
Distribution of adherence scores, based on best possible 24-h urine collection following dietary advice. Scores range from − 5 (minimum) to + 5 (maximum). For details, see text

Fifty-seven (76.0%) of stone formers delivered one, 13 (17.3%) two, 4 (5.3%) three, and 1 (1.4%) four follow-up urine collections until they reached their best adherence score. The time to reach this was 3–4 months in the majority of stone formers who had only one follow-up urine after basal evaluation and dietary advice, whereas it took 15 months for the single patient who had to repeat urine collections four times to achieve the best possible adherence score. Figure 2 presents the distribution of adherence scores among the 75 stone formers. Sixty-seven stone formers (89.3%) showed a positive value of the score (at least + 1), indicating some improvement in urinary stone risk factors (at least three out of five parameters improved).

**Fig. 3:**
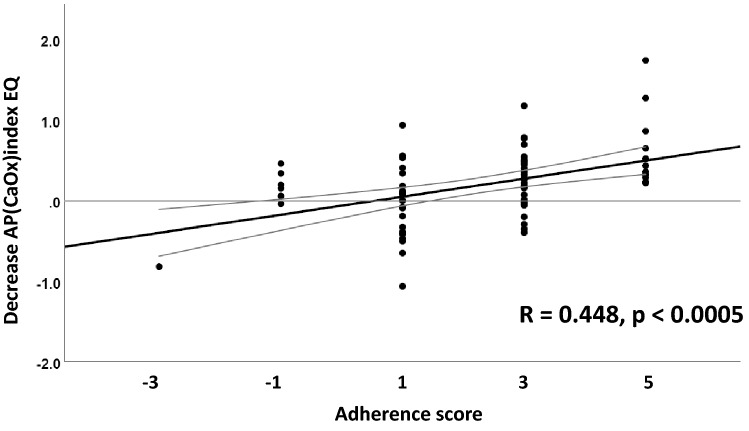
Positive linear correlation of the decrease in AP(CaOx) index EQ with adherence scores, *R* = 0.448, *p* < 0.0005, in 75 idiopathic calcium oxalate stone formers (due to data overlapping, the graph does not depict 75 single data points). For details, see text

Figure 3 depicts the significant positive correlation of decreases in AP(CaOx) index EQ after DA with adherence scores (*R* = 0.449, *p* < 0.0005). According to the data points shown, all stone formers with an adherence score of + 5 reduced their AP(CaOx) index EQ (always lower after dietary advice).

## Discussion

The most relevant finding of this study is that simple DA, if followed consequently, can reduce urinary CaOx-SS in ICSF under everyday practical conditions. Our advice comes close to what has been named empiric dietary therapy [23]. We addressed five easily measurable urine parameters, i.e., (1) increasing urine volume by increasing fluid intake, (2) reducing U-Ox by increasing calcium intake to not more than 1200 mg/day [20] simultaneously with all meals/snacks, whereby (3) U-Ca is expected to rise. Furthermore, we advised on (4) reducing “acid” intake (meat protein) and thereby U-UA as well as (5) increasing alkali consumption (vegetables/salad/fruit) and thereby U-Cit. Overall, our intervention changed all five urine parameters in the intended direction. This was accompanied by a 21.5% reduction in CaOx-SS.

As already demonstrated in another group of calcium stone formers under everyday conditions [9], increases in U-Ox and reductions in urine volume emerged as the most important determinants of a rise in CaOx-SS. On the other hand, CaOx-SS in the present study fell despite a significant 47% increase in calcium excretion, and changes in CaOx-SS after DA were not at all correlated to changes in U-Ca. This is again in complete accordance with our previous supersaturation calculations [9], using EQUIL 2 [11]. For many years, there has been a debate about the relative effects of U-Ca and U-Ox on urinary CaOx-SS. Based on physico-chemical principles, Robertson et al. [12] have convincingly demonstrated years ago that CaOx-SS in urines exponentially rises with U-Ox concentrations over the full concentration range occurring in humans, i.e., up to 0.7 mmol/L. On the other hand, CaOx-SS plateaus if U-Ca concentrations rise above 5 mmol/L even beyond the normal range, i.e., up to 15 mmol/L [12]. Opposite to these results, Pak et al. [24] have provided evidence that increasing urinary concentrations of both calcium and oxalate exert similar effects on urinary CaOx-SS. However, their calculations were performed only at the lower urinary concentration ranges of calcium (up to 7.5 mmol/L) as well as oxalate (0.46 mmol/L) [24], Fig. 1, values that could be exceeded at least transiently in human beings. Indeed, the ingestion of 50–100 g of chocolate can induce urinary oxalate excretion rates to reach values usually found in cases with primary hyperoxaluria [25]. Altogether, these findings emphasize the crucial role of increases in U-Ox for urinary CaOx-SS, whereas U-Ca is rather of negligible importance in contributing to CaOx-SS under everyday practical conditions.

Our strategy to reduce intestinal oxalate absorption by simultaneously ingesting calcium with all meals/snacks and thereby avoiding increases in U-Ox is based on previous investigations by ourselves [13], mentioned above, as well as by others [26, 27]. When calcium carbonate was administered as a supplement, either 1 g with every meal or 3 g in one dose at bedtime, to healthy volunteers on a controlled diet, calciuria rose to a similar extent under both conditions in comparison with baseline values [26]. However, urinary oxalate excretion as well as CaOx-SS, determined by Tiselius’ index as in our study, only increased when calcium supplements were taken separately from meals at bedtime, whereas it fell significantly when calcium supplements were taken simultaneously with meals [26], as in the present study. Presumably, ingestion of calcium supplements with meals reduced oxalate absorption and thereby abolished subsequent rises in U-Ox [26]. Indeed, in a tightly controlled study, Holmes et al. [27] had demonstrated that urinary oxalate excretion was determined by the oxalate/calcium ratio in the diet: both an increase in oxalate consumption at stable calcium intake as well as a reduction in calcium intake at stable oxalate consumption raised urinary oxalate excretion, indicating that the amount of dietary calcium in relation to the amount of ingested oxalate affects the intestinal bioavailability of oxalate [27].

It is well known that an exaggerated animal protein intake (meat, fish, poultry) is associated with increases in U-Ca, U-Ox and U-UA, whereas decreases in U-Cit and urine pH are observed [4]. Our advice, aiming at an improved equilibrium between “acid”—only one daily serving of animal protein—and “alkali”—at least three daily servings of vegetables/salad/fruit—was able to significantly reduce U-UA (marker of “acid”) and tendentially increase U-Cit (marker of “alkali”). As explained above (see results), the latter was due to the fact that the overall protein intake, i.e., animal as well as dairy protein, was unchanged, when assessed by urinary urea excretion, because we had advised to increase calcium intake also from dairy products, which carries an extra acid load from dairy protein [22].

An advantage of our approach is that the maximum adherence score of + 5, i.e., changes of all five relevant urine parameters (volume, U-Ca, U-Ox, U-UA and U-Cit) in the intended direction, predicted a reduction in CaOx-SS in 100% of cases (see Fig. 3). If confirmed in higher numbers of ICSF, the use of this easily derived adherence score could be a clinical surrogate for more sophisticated supersaturation calculations in the future.

The study has some limitations: first of all, the individualized DA, although based on simple principles, is relatively time consuming to perform and might, therefore, not be suited to any kind of stone clinic, for instance in situations with time restrictions due to heavy patient load. In addition, motivating patients again and again to re-adapt their diet and collect control urines several times until they reach a satisfactory result may not always be easy. Second, a single 24-h urine as a control of good adherence to DA may be insufficient. Indeed, our previous study has demonstrated that self-declared good adherence to dietary and lifestyle measures (100% adherence on 6–7 weekdays) was only reported by 26.1% of stone formers after 3 months [3]. Thus, dietary patterns may differ from day to day and—at least in some patients—only be “ideal” during stone clinic-ordered 24-h control urine collections. Third, the strategy is not generalizable and for instance would have to be thoroughly tested in a cohort of the increasingly common calcium oxalate monohydrate stone formers with enteric hyperoxaluria after bariatric surgery [28]. And last but not least, reducing supersaturation within 3–4 months after DA does not automatically imply a long-term reduction in stone recurrence rate.

In conclusion, our simple DA addressing an increased fluid intake, raising calcium consumption with all nutrients to reduce intestinal oxalate absorption, and balancing the acid–base content of the diet can reduce urinary CaOx-SS in ICSF by 21.5%. We provide again evidence that CaOx-SS in urine as driving force for calcium oxalate stone formation is primarily driven by increases in U-Ox and decreases in urine volume, but not by the extent of calciuria. By considering changes of five 24-h urine parameters after dietary advice—volume, U-Ca, U-Ox, U-UA and U-Cit—a simple adherence score can be calculated which significantly correlates with alterations in CaOx-SS. In the future, this might help physicians to evaluate adherence to dietary advice and to individually tailor recommendations to idiopathic calcium stone formers, especially in settings where supersaturation calculations are not routinely obtained.
